# Evolutionary cell biology traces the rise of the exomer complex in Fungi from an ancient eukaryotic component

**DOI:** 10.1038/s41598-018-29416-4

**Published:** 2018-07-24

**Authors:** Inmaculada Ramirez-Macias, Lael D. Barlow, Carlos Anton, Anne Spang, Cesar Roncero, Joel B. Dacks

**Affiliations:** 1 Department of Cell Biology, Faculty of Medicine and Dentistry, University of Alberta, Edmonton, Alberta Canada; 2 Instituto de Biología Funcional y Genómica (IBFG) and Departamento de Microbiología y Genética, CSIC-Universidad de Salamanca, 37007 Salamanca, Spain; 3 Biozentrum, University of Basel, Basel, Switzerland

## Abstract

Cargo is transported from the *trans*-Golgi Network to the plasma membrane by adaptor complexes, which are pan-eukaryotic components. However, in yeast, cargo can also be exported by the exomer complex, a heterotetrameric protein complex consisting of two copies of Chs5, and any two members of four paralogous proteins (ChAPs). To understand the larger relevance of exomer, its phylogenetic distribution and function outside of yeast need to be explored. We find that the four ChAP proteins are derived from gene duplications after the divergence of *Yarrowia* from the remaining Saccharomycotina, with BC8 paralogues (Bch2 and Chs6) being more diverged relative to the BB8 paralogues (Bch1 and Bud7), suggesting neofunctionalization. Outside Ascomycota, a single preduplicate ChAP is present in nearly all Fungi and in diverse eukaryotes, but has been repeatedly lost. Chs5, however, is a fungal specific feature, appearing coincidentally with the loss of AP-4. In contrast, the ChAP protein is a wide-spread, yet uncharacterized, membrane-trafficking component, adding one more piece to the increasingly complex machinery deduced as being present in our ancient eukaryotic ancestor.

## Introduction

Trafficking through an elaborate set of endomembrane organelles allows for spatially distributed and compartmentalized chemical microenvironments that are required for proper tissue and cellular function in eukaryotes. It is crucial for normal cellular activity, with dysregulation of membrane-trafficking being implicated in a wide range of human diseases^1–3^. As a feature of all eukaryotic cells, membrane-trafficking is also heavily implicated in the pathogenic mechanisms of a wide range of microbial organisms that impact our health and livelihoods. The process of membrane trafficking distributes material throughout the cell and mediates its release or internalization from the extracellular space. Such internalization is undertaken by the endocytic pathway, while release of material and presentation on the cell surface is mediated by the secretory pathway^4^.

This secretory pathway consists of distinct steps for anterograde and retrograde transport between the membrane compartments. Transport from the endoplasmic reticulum to the plasma membrane (PM) via various intracellular organelles is known as anterograde transport^5^. The *trans-*Golgi Network (TGN) is the penultimate compartment in anterograde transport for most secreted proteins, with traffic from the TGN to the PM being the final step in the process.

**Figure 1:**
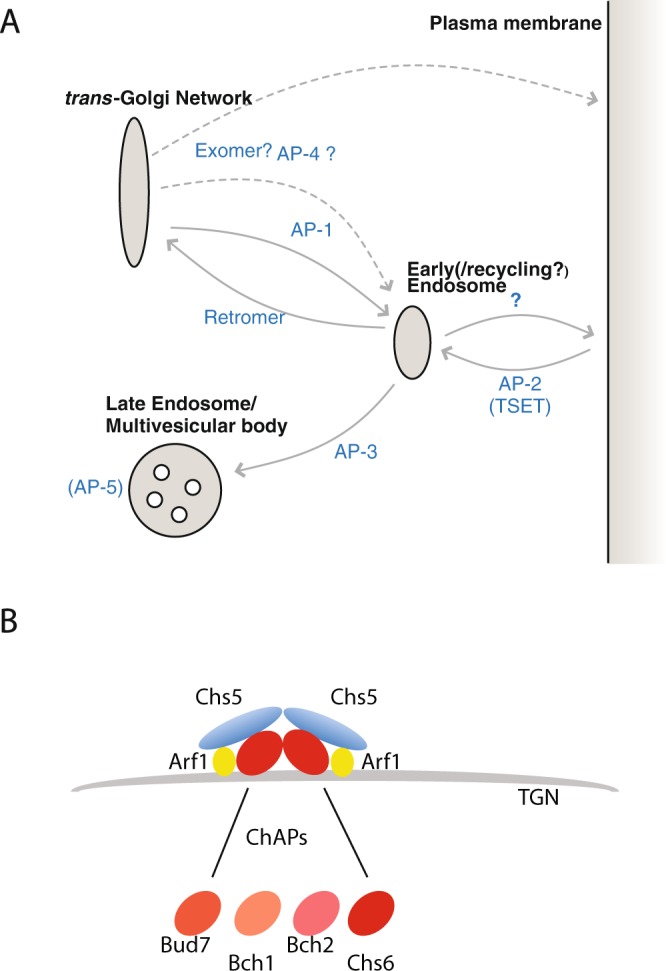
Membrane-trafficking pathways and the exomer complex. (**A**) Schematic of membrane trafficking pathways between the *trans-*Golgi Network and plasma membrane identified in eukaryotes. (**B**) Schematic of exomer components as characterized in *S*. *cerevisiae*. Exomer consists of a homodimer of Chs5, and each of the Chs5 has one binding site for a ChAP (Bud7, Bch1, Bch2, Chs6). Different combinations of ChAPs can bind to Chs5.

Extensive cell biological work in animal and fungal model systems has identified a sophisticated set of molecular machinery responsible for membrane trafficking^4,6^. Selection of cargo for inclusion into transport vesicles between organelles within the late secretory and endocytic system is performed by a related set of hetero-tetrameric protein complexes called the Adaptor Protein (AP) complexes. During transport from the TGN to the PM, cargo can use different routes (Fig. 1A). The cargo may traffic to the lysosome, mediated by the AP-3 complex^7,8^. Alternately it can go directly from the TGN to the PM (with or without endosomal intermediates) mediated by AP-1 or AP-4 complex binding^9^.

Besides these pathways, cargo can be exported from the TGN to the PM in an AP-independent manner. One such example is the exomer-mediated pathway^9^. Exomer was discovered in yeast as being involved in the transport of chitin synthase Chs3 to the plasma membrane^10,11^. It is an Arf1 GTPase-dependent protein complex^10–12^, consisting of two copies of the core protein Chs5, and any two members of four paralogous proteins known as the ChAPs (for Chs5 and Arf1 binding Proteins): Chs6, Bud7, Bch1 and Bch2^10–13^ (Fig. 1B). Theoretically, ten distinct variants of exomer tetramers can exist, because different combinations of ChAPs can bind to the Chs5 dimer, but only a subset of all possible complexes is probably ever formed in the cell *in vivo*^14^. Although the ChAPs are homologous proteins, they vary in their contribution to exomer assembly^14^.

To date less than a handful of bona fide exclusively exomer-dependent cargoes have been reported (Chs3, Fus1, Pin2)^10,15,16^, none of which are conserved in mammalian cells. More recently, however, this exclusivity of exomer cargo selection was questioned as some plasma membrane proteins may use either the conventional or the exomer-dependent route^17^. The ion transporter Ena1 is such a protein, because exomer-dependent polarized localization of Ena1 is necessary to counteract toxic cation concentrations^17^, and Ena1 is conserved from yeast to mammals.

Exomer has thus far only been characterized in yeasts^18^. Membrane-trafficking, however, is a feature of all eukaryotic cells. A growing body of molecular evolutionary studies has demonstrated the conservation across eukaryotic diversity of most of the major protein families implicated in membrane trafficking^19^. This means that the Last Eukaryotic Common Ancestor (LECA) possessed a sophisticated endomembrane complement. These studies also provide a context for which aspects of cell biological models of membrane-trafficking, derived from work in animals and fungi, are broadly applied to eukaryotic cells in general. The APs and their related complexes (COPI, TSET) have been shown as ancient features of the eukaryotic cell^20–23^. However, several of these complexes (AP-4, AP-5, TSET) have also been shown to be lost repeatedly in the course of eukaryotic evolution^19,23^, and additional lineage specific complexes derived from APs (eg. GGAs, Stonins) have also been identified^24–26^. Indeed, the possibility has been raised that exomer may be homologous to the ear domain of the large subunit of Adaptins or other trafficking complexes^12^. A previous report noted the presence of ChAP proteins in ciliates and red algae and raised the hypothesis that exomer was an ancient feature of the eukaryotic trafficking system^10^. However, this was based on limited genomic sampling, over a decade ago. A more systematic examination of exomer component distribution and phylogeny is needed.

In this study, we perform an extensive evolutionary analysis of the exomer complex, to better understand its history and the extent to which exomer is a feature of the general eukaryotic membrane-trafficking complement. We also investigate the functional homology of exomer components through microscopy and heterologous complementation, in order to assess exomer relevance in diverse fungi.

## Results

### Bch1, Bch2, Chs6, Bud7 are Saccharomyces-specific proteins but the exomer complex is likely present across the Fungi

**Figure 2:**
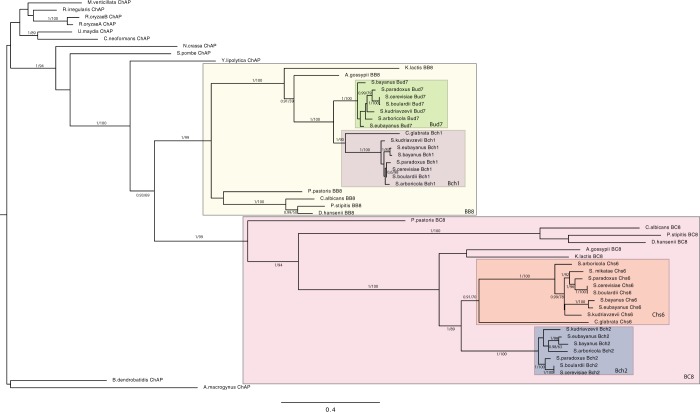
Exomer components and their relationship in Fungi. Phylogenetic tree showing the duplication of ChAP components during Saccharomycotina evolution from a pre-duplicated ChAP protein to the common ancestor of Bch1/Bud7 and common ancestor of Bch2/Chs6, followed by the duplication giving rise to these individual components prior to *Saccharomyces* speciation. Smaller internal boxes denote the four near *Saccharomyces*-specific proteins (Green box: Bud7 clade: Purple box: Bch1 clade; Orange box: Chs6 clade; Blue box: Bch2 clade), while the outer boxes denote the clades of BB8 (i.e. Yellow box: Bch1, Bud7 and preduplicated homologues) and BC8 (Pink box: Bch2, Chs6 and preduplicated homologues) respectively. The MrBayes topology is shown with posterior probability values (first number) and ML bootstrap support values (second number) overlain on all nodes reconstructed with 0.8PP (Bayesian posterior probabilities) and 50% or better. Scale bar: Number of changes or substitution per site.

Exomer subunits have been best characterized in *Saccharomyces cerevisiae*, but also reported in *Schizosaccharomyces pombe*^18^, and noted to be present in various fungal genomic databases. In order to more systematically investigate the presence and history of exomer subunits outside of *S*. *cerevisiae* and other Ascomycota, we used comparative genomics to search diverse eukaryotic genomes. Beginning with the Ascomycete fungi, we found candidate orthologues of the four related ChAP proteins (Bud7, Bch1, Chs6, Bch2) and phylogenetic analysis was performed to assess their distribution and inter-relation (Fig. 2). With very robust support, we find that Bch1 and Bud7 are the result of a specific duplication (node uniting the green and purple boxes: 1.00/100% posterior probability/bootstrap support) very near the divergence of the Saccharomyces, as are Bch2 and Chs6 (node uniting the orange and blue boxes clades-1.00/89%). Furthermore, the gene duplication giving rise to these two larger encompassing clades (node uniting the yellow and pink boxed clades-0.93/69%) likely took place after the divergence of *Yarrowia lipolytica* with the rest of the Saccharomycotina. To more easily refer to these clades, we discuss the clade of Bud7 plus Bch1 and its preduplicate paralogues as BB8 (Bch1 + Bud7 = BB8) while Bch2, Chs6 and the preduplicate paralogues are referred to as BC8 (Bch2 + Chs6 = BC8).

**Figure 3:**
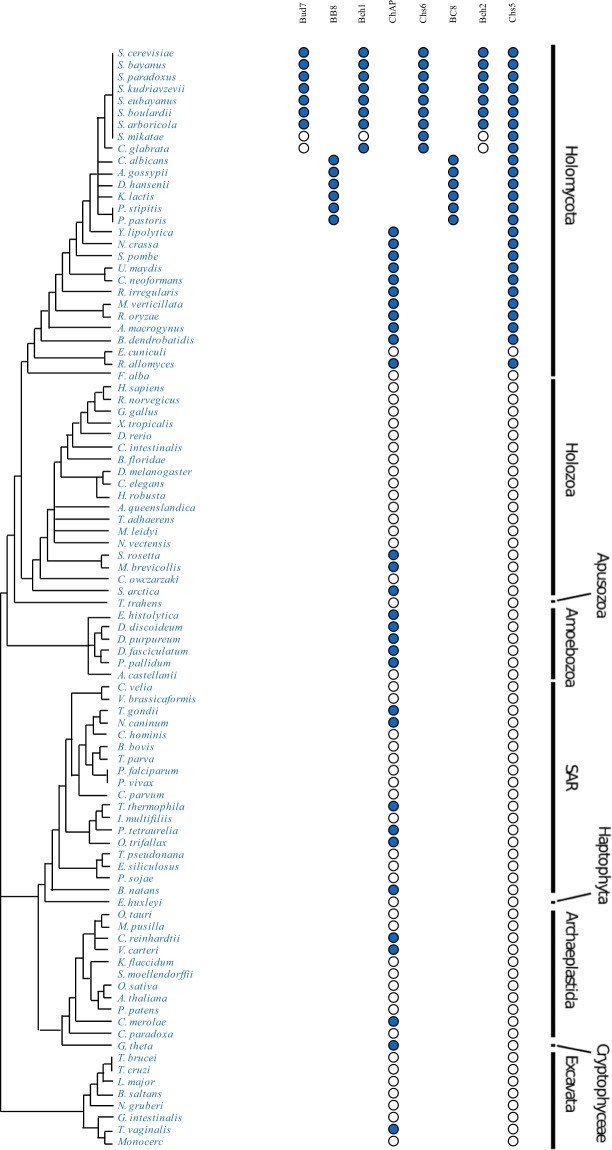
Dot plot of exomer orthologues. This diagram illustrates the data from homology searching (Supplementary Table S1) and supported by phylogenetic analysis (Fig. 2) showing the presence of duplicated ChAP components in the Saccharomycotina and the presence of a pre-duplicated ChAP protein in diverse eukaryotic lineages, as well as Chs5 as a pan-fungal component. Filled dots indicate the identification of an orthologue while empty indicates that no orthologue was found. **Relationships shown are based on^52–54^.

Expanding the search outside the Ascomycota identified a single homologue related to the four *S*. *cerevisiae* ChAP proteins, referred to simply as ChAP, present in nearly all Fungi (Fig. 3, Supplementary Table S1). Searches for Chs5 similarly revealed robust orthologues in the diversity of fungal genomes. We speculate the presence of an exomer complex and exomer-trafficking pathways in Fungi, based on the presence of the central Chs5 protein and homodimer of the ChAP proteins.

In support of the prediction of a functional exomer complex in Fungi, at least 46 of the 69 exomer subunits identified in our homology searches were listed as having gene expression support in their respective genomic databases (Supplementary Table S1). This expression data inherently supports the genes as valid and not merely pseudogenes or erroneous gene predictions (Supplementary Table S1).

### The initial duplication of ChAP allowed for neofunctionalization in Fungi

Having detailed the ChAPs complement in Fungi and clarified the timing of the duplications, we wanted to better understand the functional evolution amongst ChAP protein paralogues. Upon gene duplication, several fates are possible including separation of ancestral function to the paralogues (i.e. subfunctionalization) or retention of ancestral function in one paralogue and gain of novel function (i.e. neofunctionalization) in the other^27^. These different options are manifested as sequence divergence from the pre-duplicated proteins. We assume that equal divergence between paralogue sets is the result of subfunctionalization or simply accumulation of changes due to neutral evolutionary processes, while unequal divergence between lineages suggests neofunctionalization. We assessed the rate of change in ChAP proteins in two ways. Firstly, it is apparent from the branch lengths observed in Fig. 2 that the paralogues within the BC8 clade (pink box) are more divergent than are the paralogues within the BB8 clade (yellow box) suggesting that neofunctionalization and retention had taken place in the two respective clades.

**Figure 4:**
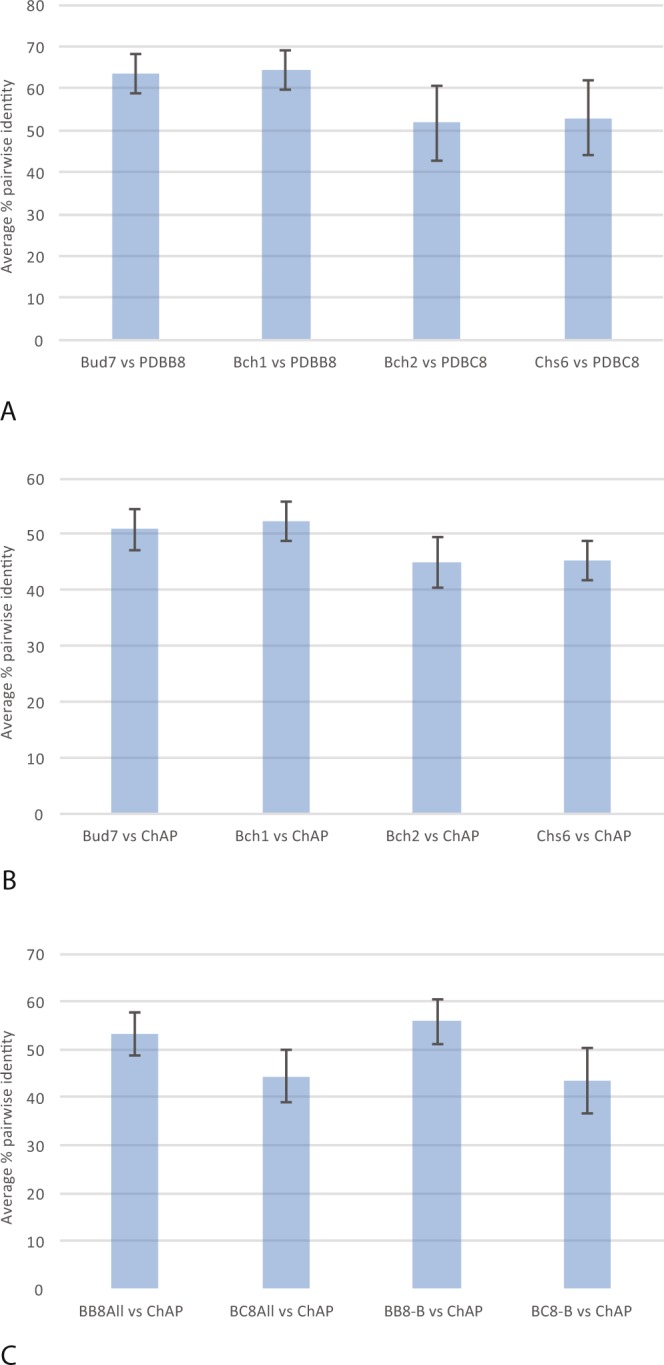
Percent pairwise identities of ChAP proteins across Fungi. (**A**) Comparisons of each *Saccharomyces*-specific proteins versus the preduplicate genes from the BB8 and BC8 clades. (**B**) Comparisons of each *Saccharomyces*-specific protein versus preduplicate ChAP genes from non-ascomycete fungi. (**C**) Comparisons of all BB8 and all BC8 versus the preduplicate ChAP genes from non-ascomycete fungi followed by BB8 and BC8 balanced to have a single ascomycete genus represented. This final comparison is the only one with non-overlapping errors bars, showing a robust difference in values. Error bars show one standard deviation of the average percent ID in all pairwise comparisons between the sets. The taxon sets were defined according to the Annotation labels in Supplementary Table S1 and defined as follows. Bud7: Bud7Spar, Bud7Scer, Bud7Sbou, Bud7Sbay, Bud7Seub, Bud7Sarb. Bch1: Bch1Sbay, Bch1Seub, Bch1Skud, Bch1Sarb, Bch1Spar, Bch1Scer, Bch1Sbou. Bch2: Bch2Scer, Bch2Sbou, Bch2Spar, Bch2Skud, Bch2Sarb, Bch2Sbay, Bch2Seub. Chs6: Chs6Sbay, Chs6Seub, Chs6Sarb, Chs6Skud, Chs6Smik, Chs6Scer, Chs6Sbou, Chs6Spar. PDBB8: BB8Ppas, BB8Calb, BB8Dhan, BB8Psti, BB8Klac, BB8Ago. PDBC8: BC8Klac, BC8Cgla, BC8Agos, BC8Psti, BC8Ppas, BC8Calb, BC8Dhan. ChAP: ChAPYlip, ChAPNcra, ChAPAmac, ChAPBden, ChAPSpom, ChAPCneo, ChAPUmay, ChAPMver, ChAPRirr, ChAPRory. BB8All: The set of Bud7 + Bch1 + PDBB8. BC8All: The set of Bch2 + Chs6 + PDBC8. BB8-B: The set of PDBB8 + Bud7Scer + Bch1Scer. BC8-B: The set of PDBC8 + Bch2Scer + Chs6Scer.

Secondly, because phylogenies are based only on the positions that are homologous to all genes considered and so some sequence data are excluded from the analysis, we calculated all possible pairwise percent sequence identities between the various ChAP proteins. Using these percentages, we explicitly tested the hypothesis that either Bud7 or Bch1 had neofunctionalized relative to the other by assessing whether either sets had higher percent identity to the preduplicate BB8 paralogues. Likewise, we explicitly tested the hypothesis that Bch2 or Chs6 had neofunctionalized by assessing whether either set had higher percent identity to the preduplicated BC8 genes. As seen in Fig. 4A, none of these comparisons were strongly different. We also tested the idea that one of Bch1, Bch2, Bud7 or Chs6 was significantly less diverged from the preduplicate ChAP sequences. Again, we found none of the *Saccharomyces*-specific protein sequences to show a higher percent identity to the preduplicate ChAP proteins than any of the others (Fig. 4B). However, consistent with the observations from the phylogeny, the sequences from the BC8 clade appeared to be more diverged than those of the BB8 clade. When considering all sequences, this was not outside the standard deviation of the average pairwise identity (Fig. 4C). However, when we accounted for the over-representation of *Saccharomyces* sequences (that would be counted as individual data points but yield nearly identical values and thus bias the calculation), by including only the *S*. *cerevisiae* sequences, we found that the BC8 sequences were more diverged (BB8 = 55.84% +/− 4.6 vs BC8 = 43.34% +/− 6.8), outside of one standard deviation (Fig. 4C). These analyses, and the assumption that sequence divergence reflects functional divergence, all suggest that the first gene duplication that gave rise to the BB8 and BC8 clades resulted in a neofunctionalization of the BC8 paralogues. The BB8 genes appear to be less diverged and thus are predicted to be more like the ancestral ChAP protein.

### ChAP, but not exomer, is a widespread feature of the eukaryotic membrane-trafficking complement

The presence of exomer subunits in Fungi and earlier reports of exomer subunits in Mycetozoa, red algae and ciliates^10^ prompted us to search for homologues in the rest of eukaryotes. Despite finding common signatures for BRCT and FN3 domains (see below), we could find no credible orthologues of Chs5 in diverse eukaryotes outside of the Fungi, using BLAST and HMMER homology-searching methods.

By contrast, we were able to identify ChAP orthologues in genomes of organisms from across the breadth of eukaryotes (Fig. 3). The pattern of a broad evolutionary distribution, but frequently with only selected organisms possessing at least one ChAP protein, was further confirmed by searches that used the relevant member of the supergroup as the target database for reciprocal best hit validation. We suggest that the ChAP protein is a component of the trafficking system of diverse eukaryotes, and was present in the LECA, but may be expendable and has been lost on many occasions, including at the multicellular boundary in the independent lineages giving rise to animals and plants.

Finally, in an effort to identify the origins of the exomer complex, we took advantage of the sensitivity afforded by HMM-based searching methods for all of the homology searches that we performed. In cases when exomer homologues were identified, we tallied the functional annotation of the next best-scoring, non-orthologous protein. In cases when no clear exomer candidate was retrieved (i.e. the top hit had a robust functional annotation as a different protein), we tallied that annotation. We were not able to find any evidence of specific homology between a ChAP and a particular protein, nor any for Chs5 (data not shown). We further performed HMMER searches into the metagenomes of the Asgardarchaeota, the recently described most closely related archaeal lineage to eukaryotes with which homologues of several eukaryotic membrane trafficking machinery components are exclusively shared, implying the archael origin of this machinery^28^. However, searches into the *Candidatus Thorarchaeota archaeon AB_25*, *Candidatus Heimdallarchaeota archaeon LC_2*, *Candidatus Odinarchaeota archaeon LCB_4* and *Candidatus Lokiarchaeota archaeon CR_4* genomes yielded no significant results that met the forward and reverse BLAST criteria set out in the Materials and Methods. Although the presence of BRCT domains and TPR repeats in Chs5 and ChAP proteins, respectively, are suggestive of origins from other eukaryotic proteins containing these domains, no clear hypothesis regarding the origins of exomer components is supported at this time.

### Exomer complex validation outside Saccharomyces genus

Our informatics analyses strongly suggested the presence of an exomer complex at work along the whole fungal lineage. In order to test this hypothesis, we heterologously expressed the N-terminal part of the Chs5 genes from several fungi including the basidiomycete *Ustilago maydis* and the zygomycete *Mucor circinelloides* in *S*. *cerevisiae* cells deleted for *CHS5*. Taking into account that the N-terminal part of Chs5 (ScChs5*) is functional^29^, we amplified this fragment by PCR from *S*. *cerevisiae*, *Kluyveromyces lactis*, *Candida albicans*, *U*. *maydis* and *M*. *circinelloides* (see Supplementary Fig. S1), cloned them, fused to a GFP-tag at their C-terminus, and expressed the resulting fusion proteins under the strong pGAL1 promoter in *S*. *cerevisiae*.

**Figure 5:**
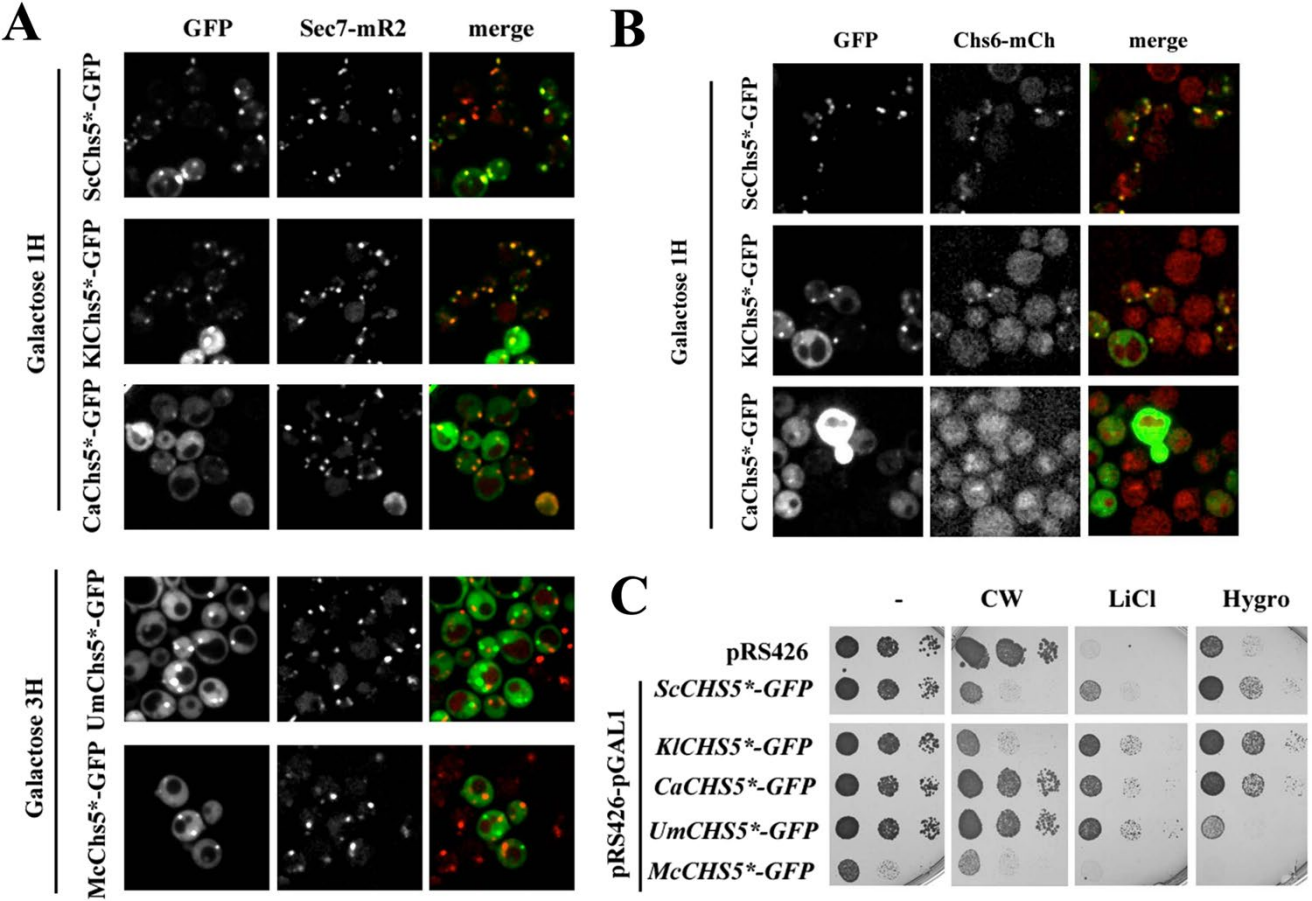
Heterologous expression of N-terminal fragments of *CHS5* from diverse fungal lineages: *S*. *cerevisiae* (ScChs5*), *K*. *lactis* (KlChs5*), *C*. *albicans* (CaChs5*), *U*. *maydis* (UmChs5*) and *M*. *circinelloides* (McChs5*). (**A**) Localization of N-terminal fragments of Chs5 from the indicated proteins tagged with GFP and co-localization of these fragments with the TGN marker Sec. 7-mRuby2. Images were acquired after induction in galactose media for the indicated times. (**B**) Co-localization of heterologously expressed versions of Chs5 with the Chs6 ChAP. The plasmids containing the indicated version of Chs5* were transformed in a *S*. *cerevisiae chs5∆* mutant with a chromosomally tagged version of *CHS6* (*CHS6-mCherry*). Proteins were expressed in galactose media for one hour as indicated. Note the apparent co-localization of green and red signals in some strains, indicative of an effective recruitment of Chs6-mCh from the cytoplasm by the heterologous expressed Chs5* fragments. (**C**) Complementation of the *chs5∆* phenotypes by the heterologous expressed proteins. Note the alleviation of some phenotypes upon overexpression on galactose media. Overexpression of McChs5*-GFP showed deleterious effects on growth, therefore the complementation test is unreliable. All test were preformed in a *S*. *cerevisiae chs5∆* strain grown overnight in raffinose media and later plated directly on galactose plates after appropriated dilution. See text and Table 1 for additional details on the interpretation of the Figure.

The localization of the proteins was assessed by fluorescence microscopy after one and three hours of expression (Fig. 5). The proteins ScChs5*, KlChs5* and CaChs5* were neatly visualized as intracellular spots after one hour, showing a strong co-localization with the TGN marker Sec. 7-mR2 (Table 1) suggestive of its correct assembly. Overexpression for three hours of both *U*. *maydis* and *M*. *circinelloides* Chs5 N-terminal fragments, lead to the formation of discrete intracellular foci that, however, did not co-localized with Sec. 7-mR2 (Fig. 5A), a result that questions the nature of these foci.

**Table 1:** Analysis of N-terminal fragments of Chs5 from different fungal origins expressed under *GAL1* promoter.

Protein	Intracellular spots after 1 or 3 hours expression	Co-Localization with Sec. 7-mR2 (%)	Co-Localization with Chs6-mCh (%)	Calcofluor sensitivity	LiCl sensitivity	Hygromycin sensitivity
ScChs5*	+++^(1h)^	93.2 ± 1^(1h)^	90.1 ± 6.2^(1h)^	++	+	+++
KlChs5*	+++^(1h)^	92.4 ± 0.8^(1h)^	52.6 ± 7^(1h)^	+	++	+++
CaChs5*	++^(1h)^	91.0 ± 0.8^(1h)^	None^(1h)^	—	++	+++
UmChs5*	+^(3h)^	<10^(3h)^	NA	—	++	—
McChs5*	+^(3h)^	<10^(3h)^	NA	NA	NA	NA

Chs5 acts as scaffold for the ChAP subunits of exomer, therefore we next determined the capacity of these N-terminal fragments to recruit the Chs6 ChAP (Fig. 5B). The ScChs5* and KlChs5* proteins co-localized neatly with Chs6-mCh after one hour in galactose in intracellular spots. However, co-localization between CaChs5* and Chs6 was almost negligible despite the correct localization of the CaChs5* at the TGN. Longer overexpression of this protein partially colocalized with Chs6 at an uncertain intracellular localization (not shown). The co-localization between UmChs5* or McChs5* and Chs6 is irrelevant since it would occur at an incorrect localization. Altogether, these results (Table 1) indicate that only the N-terminal fragments from Saccharomycotina strains assembled at the TGN and are able to recruit to some extent the Chs6 ChAP.

We next addressed the functional complementation of these proteins. Overexpression of ScChs5* complemented the *chs5∆* associated phenotypes^29^, (Fig. 5C) including calcofluor resistance, and lithium and hygromycin sensitivities. The KlChs5* fragment had a modest effect on calcofluor sensitivity but clearly alleviated lithium and hygromycin sensitivities. CaChs5* improved lithium and hygromycin resistance while was unable to restore calcofluor sensitivity. UmChs5* ameliorated only the growth on lithium plates. McChs5* overexpression results are difficult to interpret because the deleterious effects on the construct upon overexpression (notice its low growth in control plates).

In summary, most of the heterologously expressed N- terminal fragments of Chs5 were able to partially complement the *chs5∆* associated phenotypes in *S*. *cerevisiae*. Although function likely diverges the more distantly related taxa are from *S*. *cerevisiae*, these data do suggest a role for the exomer complex within fungi.

## Discussion

In *S*. *cerevisiae*, the exomer complex serves as a potential adaptor complex for the export of cargo from the TGN to the plasma membrane. In fact, there is no single exomer complex, but several different ones can be formed, presumably aiding the transport of specific cargoes. We sought to understand the extent to which this secretory pathway was present in eukaryotes beyond this model system, as well as to understand the evolutionary history of the complex.

We were able to confirm that the four proteins Bch1, Bch2, Chs6 and Bud7 are specific to the Saccharomyces (plus *C*. *glabrata*)^10^. These were produced by three gene duplications, two in the Saccharomyces (plus *C*. *glabrata*) and one after Yarrowia split from the remaining Saccharomycotina, producing a paralogous protein family from a single primordial ChAP protein. Notably, the phylogeny suggests that the duplication of the relevant paralogues took place prior to the separation of *C*. *glabrata* from the Saccharomyces. In this case, the *C*. *glabrata* Bud7 and Bch2 paralogues were subsequently lost. This would be consistent with the loss of new paralogues early after duplication and before neofunctionalization has been established. *C*. *glabrata* has also been recently noted for its genomic plasticity^30^ and so the loss of paralogues is not unreasonable.

The Chs5 protein was found to be present in all fungal genomes sampled except *E*. *cuniculi*, a member of the microsporidia that is well-established to have a reduced cellular configuration and genome complement^31,32^, including the presence of a single AP complex^33^. Based on the composition of the exomer complex as two Chs5 peptides and dimer of any of the four ChAP proteins, we speculate the presence of exomer-related pathways in all Fungi. This builds on earlier informatics work noting the presence of exomer subunits^10^, but refutes the idea that any given ChAP protein is the most ancient member of the family. Instead, our data is consistent with the initial duplication giving rise to the BB8 vs BC8 clade was the definitive event, with potential neofunctionalization occurring in the BC8 clade.

The analysis of sequence data clearly suggests an evolutionary conservation of the exomer machinery deep within the Fungi. However, the phenotypes associated with the absence of exomer are difficult to assess in fungi other than *S*. *cerevisiae*, since the exomer mutants of *U*. *maydis*, *C*. *albicans* or *S*. *pombe*^18,34^ did not show the phenotypes associated to the absence of exomer in *S*. *cerevisiae*^34^, i.e. calcofluor resistance or alkali metal cation sensitivity. In order to solve this conundrum, we opted for the heterologous expression and characterization in *S*. *cerevisiae* of the orthologues of Chs5 from diverse fungal lineages.

Our results indicate a limited capacity of the N-terminal fragments of Chs5 from different origins for their assembly in the cytosol of the yeast cells based on their intracellular localization, their ability to recruit the ChAP Chs6 and their capacity to complement some of the exomer functions in the *chs5∆* mutant. The KlChs5* protein, like the original ScChs5*^12,29^, seemed to be able to assemble a functional exomer in *S*. *cerevisiae* based on the complemented phenotypes, in clear agreement with the close evolutionary relationship between these two fungi. Apparently, the KlCsh5* fragment can serve as an efficient scaffold for the recruitment of the multiple ChAPs present in *S*. *cerevisiae*. By contrast, as observed in the co-localization experiments with Chs6, CaChs5* is less efficient for the recruitment of the ChAPs, being unable to complement the calcofluor resistance of the *chs5*∆ mutant. This result is not surprising, considering that the involvement of exomer in chitin synthesis is a recent evolutionary acquisition within the *Saccharomyces* genus associated to the functional specialization of the Chs6 ChAP^34^. Moreover, it is consistent with the neofunctionalization occurring in the BC8 ChAP clade suggested by the bioinformatics analysis. However, we have not been able to show a functional conservation for Chs5 outside Saccharomycotina since UmChs5* and McChs5* did not assemble at the TGN and do not have the ability to recruit Chs6. The capacity of UmChs5* to complement the lithium sensitivity of the exomer mutant could suggest a conserved capability that needs to be confirmed with other methods.

A common trait for exomer cargoes appears to be the polarized localization at the plasma membrane^17^, which is maintained by a delicate balance of exo- and endocytosis requiring exomer being in control of TGN export. Overall, our data suggest functional homology of exomer proteins across Saccharomycotina, while also showing differences consistent with the wide evolutionary distance of the taxa in question that probably reflect also the progressive neofunctionalization of ChAPs in the BC8 clade that led to the unique exomer specialization as a cargo adaptor in *Saccharomyces*.

We were further able to identify orthologues of a ChAP protein in a wide-range of eukaryotic organisms. However, we were also frequently unable to identify a homologue, even when searching genomic contigs and using the closest related organism as a comparison database. It is important to acknowledge the caveats of negative data in studies such as these. Failure to identify an orthologue could be due to incomplete genomic databases, and/or divergence of the protein sequence beyond detection of the algorithms. For any given search, we are conservative in our statement of “not identified”, rather than the protein not being encoded. Nonetheless, in cases when no orthologues could be identified in coherent taxonomic blocks, despite our use of the most sensitive available sequence homology searching methods, some conclusions can be drawn.

The presence of a putative ChAP, in the apparent absence of its interacting partner Chs5, raises at least two functional possibilities. On the one hand, ChAP could be interacting with an analogous subunit. On the other hand, all of the ChAP subunits in yeast are known to bind the Arf GTPase directly^10,12^. Arf is an ancient central component of the vesicle formation machinery^35^. It is possible that ChAP acted in the LECA to mediate Arf function and retains that function in those eukaryotes that possess it today. Regardless of how ChAP functions, its presence and expression indicates an additional facet to the trafficking pathways of microbial eukaryotes with medical and global health relevance, such as *Entamoeba histolytica* and *Trichomonas vaginalis* and in protists with ecological importance such as ciliates and green algae.

This is particularly true of the Fungi. Given the environmental importance of Fungi in healthy ecosystems and bioremediation, the presence of exomer genes encoded in fungal genomes from across their taxonomic breadth gives insight into the potential cellular workings of a major contributor to our biosphere. While it had previously been reported that exomer subunits could not be found in the animal and plant genomes available at the time^10^, our results show this to be the case in a much wider sampling of those taxa, and more importantly show the presence of ChAP in their respective single celled-relatives, pin-pointing when the losses occurred, thus giving more confidence of true absence rather than failure to identify the orthologue. The extension of exomer as a feature of fungal cells, and not simply yeast, therefore has intriguing potential implications for new therapeutic avenues of intervention for major crop and human pathogens.

**Figure 6:**
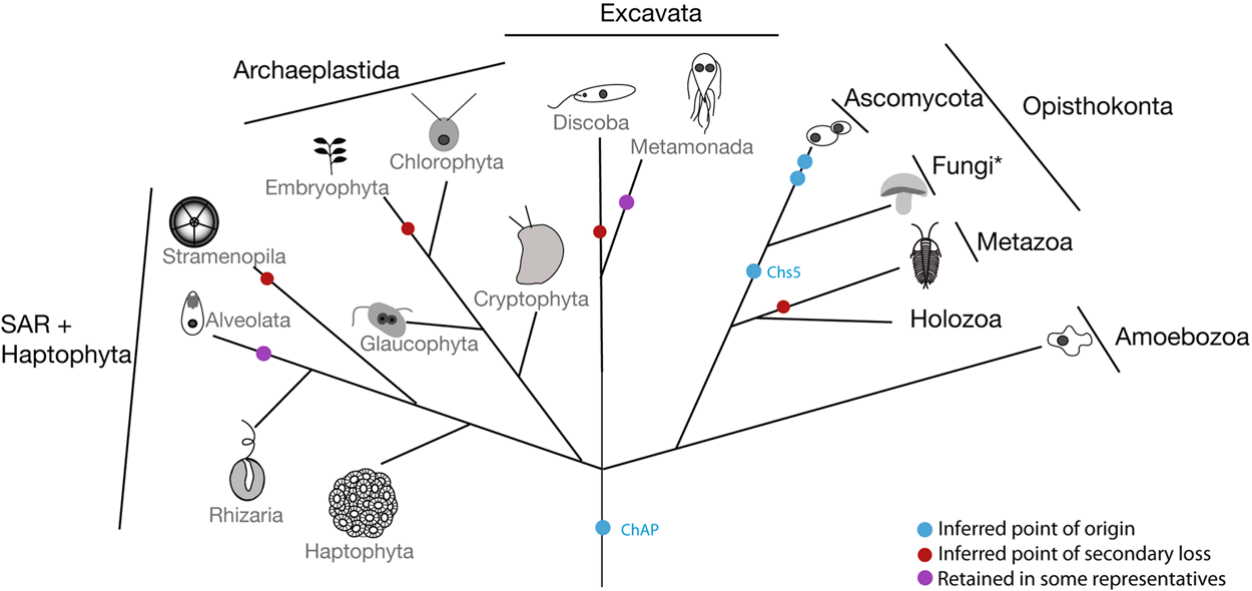
Exomer component evolution across the tree of eukaryotes. The gains and losses of exomer components are mapped on this cartoon representation of eukaryotic lineages based on presence of at least two positive orthologues in a given taxonomic group. The purple circles indicate two instances where a single representative taxon possesses the ChAP protein despite multiple other representatives lacking the gene. In both cases (*Toxoplasma gondii* and *Trichomonas vaginalis* respectively), the organisms in question are known possess more canonical or expanded eukaryotic complements and have retained aspects lost in their relatives^55,56^. The double blue circle in the ascomycetes denotes the duplications giving rise to the expanded ChAPs complement, as detailed in Figs 2 and 3. The Fungi* represents the paraphyletic assembly of Holomycota, with Ascomycota treated separately due to the additional evolutionary detail relating to exomer. Relationships are based on^57–59^.

The presence of ChAP orthologues in diverse eukaryotes means that this protein is ancient, likely present in the LECA, estimated at 1.5 billion years ago^36^. This adds yet one more component to the growing list of trafficking factors present in this apparently sophisticated ancestor^37^. The addition of the Chs5 protein in the Fungi then gave rise to what is considered to be the exomer pathway per se, which was elaborated by the gene duplications of the ChAP in the ascomycetes (Fig. 6). Intriguingly, the appearance of Chs5 in Fungi coincides with the time point at which the loss of AP-4 is first inferred to have occurred^33^. Though absent from many fungal lineages, subunits of AP-4 have been identified in basidiomycete, glomeromycete and chytridiomycete lineages, which implies that AP-4 was lost independently at least 8 times across Fungi^33^. This pattern is consistent with a reduced role for the complex and thus a relaxed selection on its presence.

AP-4 is known to mediate post-TGN trafficking and has been implicated in polarized cargo sorting in animal cells^38^. Recent data suggests that AP-4-mediated traffic proceeds via an endosomal intermediate before cargo reaches the surface^39,40^. As exomer is also responsible for post-TGN trafficking, we propose that the evolution of an exomer complex introduced redundancy in the secretory pathway that resulted in some lineages in the degeneration and loss of AP-4. Indeed, since the exomer pathway is more direct than the AP-4 pathway^39^, in cases where efficacy of the secretory route was selected, this could have facilitated AP-4 loss. Intriguingly the glomeromycete *Rhizophagus irregularis* possesses both a complete AP-4 tetramer and the Chs5+ ChAP proteins, suggesting that the two pathways can be encoded in the same organismal genome concurrently.

At the same time, our frequent failure to identify a ChAP orthologue means inferred losses in several major lineages (Fig. 6). This makes ChAP yet another example of proteins with a ‘Patchy’ distribution, as has previously been reported for other vesicle coats (AP-5, TSET), GTPases and regulators (ArfGAPC2, RabTitan)^19^. Many of these proteins, like ChAP, are present in the rest of eukaryotes but lost in animals, raising the important question of what additional cell biology exists in eukaryotes that we cannot study or detect by work in metazoan model organisms alone. New emerging model organisms from across eukaryotic diversity are becoming increasingly tractable and are yielding useful data on the cell biology of membrane trafficking (and references therein)^41^. Making better use of comparative cell biology in a representative sampling of eukaryotes should provide tremendous opportunities to explore the widespread features of the membrane trafficking system.

## Methods

### Homology Searching

Homology searches for exomer components were performed using BLASTp and HMMER homology searching in a representative sampling of eukaryotic organisms with fully sequenced genomes. *S*. *cerevisiae* sequences for Chs5, and the ChAPs Bch1, Bch2, Bud7, and Chs6 were used as initial queries. See Supplementary Table S1 for a complete list of organisms examined and the accession numbers of all sequences identified.

Initially, BLASTp (2.2.29+)^42^ searches were undertaken, using a bi-directional best hit search strategy. Retrieved proteins were deemed as positive hits, only if they were retrieved by a *S*. *cerevisiae* query and also retrieved the *S*. *cerevisiae* sequence when used as a BLAST query. An E-value cutoff of 10^−40^ was applied for Chs5 BLAST searches, in order to reduce false-positives. An inclusive E-value of 0.05 was applied to searches for the ChAP proteins. In addition, BLAST hits were required to retrieve the *S*. *cerevisiae* query with an e-value at least two orders of magnitude lower than the next best non-redundant hit.

BLAST hits that met these search criteria were further evaluated using multiple sequence alignment, and searches in the Pfam database^43^, in order to confirm similarity of their domain structure to the query proteins. Several positive BLAST hits for the ChAP proteins were found to lack ChAPs domains using these approaches, and these sequences were eliminated from the dataset.

Searches were also undertaken using HMMER (3.1b1)^44^. Positive hits from the initial BLAST analysis were aligned using MUSCLE^45^, and used to construct Hidden Markov Models (HMMs). Additional homologues identified using HMMER, were added to the HMMs for subsequent HMMER searches, until no more homologues were identified. In order to validate hits retrieved by HMMER, reverse BLAST searches were performed for each hit into the *S*. *cerevisiae* genome. HMMER hits were considered positive if the original *S*. *cerevisiae* query (or another ChAP homologue) was retrieved as the top hit in these BLAST searches. For candidate proteins that were pre-duplicates to the specific ChAP paralogues, retrieval of any ChAP query was the criteria, and then relationships were determined by phylogenetic analysis.

To reduce the risk of false negatives due to divergence between candidate orthologues and *S*. *cerevisiae*, we performed the reverse BLAST analysis for all candidate orthologues, but into organismal databases of diverse taxa from which positive orthologues had been identified in initial rounds of homology searching. If a candidate protein retrieved the identified orthologue in that organism as the top hit using the above criteria, it was also deemed a homologue. Organismal genomes used as reverse BLAST databases included, *Dictyostelium discoideum*, *Dictyostelium fasciculatum*, *Bigelowiella natans*, *Salpingoeca rosetta*, *Tetrahymena thermophila*, *Chlamydomonas reinhardtii*, *Micromonas pusilla*, *Guillardia theta*, and *Trichomonas vaginalis*.

To assess origins of the exomer components, HMMER search results for ChAPs and Chs5 were parsed for more distant homology to non-exomer components. The top non-orthologous retrieved protein, non-ChAP protein in the case of all ChAP components, was tallied for all searches and expressed as a percentage.

HMM-HMM searching was done to look for homology between exomer components and other protein families. The HHpred server: https://toolkit.tuebingen.mpg.de/hhpred was used with the following settings: Maximum of zero multiple sequence alignment generation steps (use just the input alignment), and search in all the alignment databases. The organisms for which comparisons were made were: *Arabidopsis thaliana*, *Caenorhabditis elegans*, *Drosophila melanogaster*, *Homo sapiens*, *Mus musculus*, *Plasmodium falciparum*, *Saccharomyces cerevisiae*, *Schizosaccharomyces pombe*, *Ustilago maydis*.

### Phylogenetics

Orthologues for ChAP proteins were aligned using MUSCLE v.3.8.31^45^. Alignments were visualized using Mesquite v.2.75^46^, adjusted by eye, and only regions of unambiguous homology were retained for phylogenetic analysis. All alignments are available upon request. An initial alignment of Fungal ChAP homologues of 30 taxa and 542 amino acid positions was analyzed (data not shown). A second data matrix, of 54 taxa and 395 AA positions, with several additional Saccharomyces species added and the *R*. *allomyces* sequence removed (due to its presenting a rapidly diverging sequence in the initial phylogeny) was analyzed. Bayesian phylogenetic analyses were performed using MrBayes v3.2.6^47^ with the following parameters; prset aamodelpr = mixed; mcmcngen = 10,000,000; printfreq = 10000; samplefreq = 1000; nchains = 4. Maximum likelihood analyses were performed, using RAxML version 8.2.9^48^ with the Protein GAMMA model for rate heterogeneity and the LG4X substitution matrix, obtain the optimal ML topology and bootstrap support values. 100 bootstrap pseudo-replicates were used for each analysis. Bootstrap values ≥50 were considered significant. Analyses were run on the CIPRES server^49^ and the resulting trees were visualized using FigTree v1.4.0.

### Calculation of Percent Identities between Fungal ChAP sets

For all pairwise calculations, distance matrices were calculated using FastME V2.0^50^ from an untrimmed alignment (52 taxa and 1354 position). The percent ID for each pair of sequences between the two datasets being compared was calculated using a conversion of (1 − (uncorrected p-distance) * 100). The average percent identity for comparisons between two taxon sets was calculated using the Average function in Excel and the error was calculated using the STDEV function. Note that due to the potential that the *C*. *glabrata* sequences are in fact preduplicates and phylogenetically misplaced or the alternate hypotheses that they are paralogues that have not neofunctionalized, allowing for the loss of the additional paralogue, these were treated within the preduplicate clades and not the Saccharomyces-specific ChAP clades of Bch1, Bch2, Chs6 and Bud7.

### Characterization of heterologous proteins

N-terminal fragments of Chs5 from different origins (Supplementary Fig. S1) were amplified using PCR and synthetic hybrid nucleotides that directed both amplification and recombination with the appropriated regions of the GAL promoter and the GFP in the plasmid pRS426::pGAL-CaCHS7-GFP^51^. The *S*. *cerevisiae* (ScChs5*), *K*. *lactis* (KlChs5*), *C*. *albicans* (CaChs5*) and *U*. *maydis* (UmChs5*) fragments were amplified directly from the corresponding genomic DNA, but the *M*. *circinelloides* McChs5* fragment was amplified from a cDNA gene bank kindly provided by E. Iturriaga owing to the presence of introns in this region. Amplified fragments with the right size were later co-transformed into *S*. *cerev*isiae with plasmid pRS426::pGAL-CaCHS7-GFP linearized with EcoRI and uracyl prototrophs colonies were selected. Plasmid DNA from several of these colonies was recovered in *E*. *coli*, characterized by endonuclease restriction and those showing the appropriated structure were later confirmed by direct sequencing. These plasmids were later transformed in the appropriated strains of *S*. *cerevisiae* for further experiments.

Protein localization was assessed by fluorescence microscopy using an Olympus Spinning Disk system. Cells were grown O/N in SD-Ura raffinose medium and protein expression was later induced for different times by adding galactose up to 2%. Functionality of the heterologous expressed proteins was assessed in a *S*. *cerevisiae chs5∆* mutant based on the *chs5∆* phenotypes previously described^17^.

## Electronic supplementary material

Supplementary Information

Table S1
