# Supplementary material for: Evolutionary cell biology traces the rise of the exomer complex in Fungi from an ancient eukaryotic component
Source: Sci Rep. 2018 Jul 24;8:11154. doi: 10.1038/s41598-018-29416-4 (PMC6057913; doi:10.1038/s41598-018-29416-4)
Supplement: Supplementary file 1 — Supplementary Information [file 41598_2018_29416_MOESM1_ESM.pdf]

Evolutionary cell biology traces the rise of the exomer complex in Fungi from an ancient eukaryotic component

Inmaculada Ramirez-Macias[1], Lael D. Barlow[1], Carlos Anton[2], Anne Spang[3], Cesar Roncero[2], and Joel B. Dacks[1]*

1 Department of Cell Biology, Faculty of Medicine and Dentistry, University of Alberta, Edmonton, Alberta
2 Instituto de Biología Funcional y Genómica (IBFG) and Departamento de Microbi-ología y Genética, CSIC-Universidad de Salamanca, 37007 Salamanca, Spain
3 Biozentrum, University of Basel, Basel, Switzerland

```
ScCHS5*   ---------------------MSSVDVLLTVGKLDASLALLTTQDHHVIEFPTVLLPENV
KlCHS5*   -----------------------MVEVSLTVGKVDASLAILTTADHYVIEFPTVLLPESV
CaCHS5*   -----------------------MVEVSLTVGKLDASLALLLTKDHHLIEFPTILLPNGV
UmCHS5*   MSAHYVGQIAPNYIHSTMQSNSSDDSFTFTVGKLDAGMAILIGDKASLIEFPSLLLPPGV
McCHS5*   -----------------------MTSVQFTVGKLDAGMAILLTEDHHLIEFPSLLLPKGV
                                   .. :****:**.:*:*   .  :****::*** .*

ScCHS5*   KAGSIIKMQVSQNLEEEKKQRNHFKSIQAKILEKYGTHKPESPVLKIVNVTQTSCVLAWD
KlCHS5*   EAGSVVTITVEQDLEQEIKQKKKFQSVQEQILAKYGTDPPAEPVLHLSNATQTSCVIHWD
CaCHS5*   RAGSIIKIRCDQDFDSEKEEAEKFTKIQDEILQTFATNLPQPPNLKIKNVTQTSCVLEWD
UmCHS5*   SSGSVVNIRVLRNDQQERRQKDEFDQLQEDILHTFGQQSPKPPALRLRNVTQTSVTLEWD
McCHS5*   TSGSIVNIAVSRNIPEEEKKMKEFWELQESILATFGQDEPEVPVVRVKNITQTSLILEWD
           :**::.:   ::  .* .: ..* .:* .** .:. . *  * ::: * ****  : **
                                    FN3
ScCHS5*   PLKLGSAKLKSLILYRKGIRSMVIPNPF------KVTTTKISGLSVDTPYEFQLKLITTS
KlCHS5*   PIHLGSSQLKSLILYKQGNRHQVITPGSNRDGTTDNNSLKISGLSVDFEYEFKLLLNTTS
CaCHS5*   KLNLGTATLKNLILFKDGKKLGSIPQPL------NNRTSKLSGLPIDKSFKVQLRLDTTA
UmCHS5*   RLTLATATLLSLDIYRNGQRLAPIPNPL------HNTSTKLSGLDVNAEYTFHLVLKTTA
McCHS5*   PLLLHTAKLRSLDIYKNGTKLAQH-VPS------DTNFIKLSGLDVDHEFEFHVVIKTTA
           : * :: * .* :::.* :             .    *:*** ::  : .:: : **:
                                                  BRCT
ScCHS5*   GTLWSEKVILRTHKMTDMSGITVCLGPLDPLKE-ISDLQISQCLSHIGAR-PLQRHVAID
KlCHS5*   GKFWSNTLKVHTHKMTDMSGITACVGPNTKLGDNISLDQIKRSLQNIGAK-PLQDHVTLE
CaCHS5*   GTFLSNEIEVTTHKMTDLSGITVCLGDLTPNDQ-FNKEDIEEALKNMGAKYPVQQQVKVD
UmCHS5*   GTFSSPVIKTRTHTINDTSGISVCFGHVHDAEM--L-QAAKKALTSMKAKW--HDRIQIE
McCHS5*   GEYKSNKVTARTHKMENLTGIRVAFGTFEEPEP--AITELKDLLSKMGASW--SDEVSVE
          *   *  :   **.: : :** ...*              .  *  : *      .: ::

ScCHS5*   TTHFVCNDLDNE--------ESNEELIRAKHNNIPIVRPEWVRACEVEKRIVGVRGFYLD
KlCHS5*   TTHFIATEFEDSEGV-----TSDEELAHARDNNIPIVRPEWIRACELEKRIVGVRDFYLD
CaCHS5*   TTHFLCTREN----------KQNPEYVKANDMNIPIIRPEWLKACERERRIVGVRDFYVK
UmCHS5*   TTHYVCTDPRSPGDVNAPQGSQGQGYSKAMQLSIPAVLPHWIFACHDQKKMVGISAFYLD
McCHS5*   TTHLLAQLP------------GGANYDRAVQHSIPIVKPEWLVQCDRNKKI*--------
          *** :.                 .    :* . .** : *.*:  *. ::::

ScCHS5*   ADQ--SILKNYTF*
KlCHS5*   SDP--SNFLNYKF*
CaCHS5*   DCVLPDIFAKNYWQK*
UmCHS5*   QDPPNASTIRQ*
McCHS5*   -----------
```

Figure S1. N-terminal fragments of Chs5 from different origins as indicated: *S. cerevisiae* (ScChs5*)**,** *Kluyveromyces lactis* (KlChs5*), *Candida albicans* (CaChs5*), *Ustilago maydis (UmChs5*) and Mucor circinelloides* (McChs5*). All fragments were cloned by PCR from genomic DNA, except McChs5* that was amplified from a c-DNA library, using specific primer designed to amplify from the initial Met to the last amino acid shown in the figure. Fragments were labeled in the text and figures as Chs5* to indicate that they correspond to the N-terminal region of the proteins, a region that includes the essential FN3 and BRCT domains as indicated in the Figure.
